# Olfactory and taste disorder: The first and only sign in a patient with SARS-CoV-2 pneumonia

**DOI:** 10.1017/ice.2020.151

**Published:** 2020-04-20

**Authors:** Youngeun Jang, Hyo-Ju Son, Seungjae Lee, Eun Jung Lee, Tae Hyong Kim, Se Yoon Park

**Affiliations:** Division of Infectious Diseases, Department of Internal Medicine, Soonchunhyang University Seoul Hospital, Soonchunhyang University College of Medicine, Seoul, Republic of Korea

*To the Editor—*Severe acute respiratory syndrome corona virus 2 (SARS-CoV-2), first reported in Wuhan City of Hubei Province of China, has now rapidly spread throughout the world.^1^ Genome sequencing showed that the causal agent of Coronavirus disease 2019 (COVID-19) is a β-coronavirus belonging to subgenus of severe acute respiratory syndrome (SARS) viruses but a different clade.^2^ Common clinical manifestations include fever, cough, fatigue, dyspnea, and myalgia or arthralgia.^3^ Recently, Giacomelli et al^4^ reported that 20 of 59 (33.9%) of SARS-CoV-2–positive hospitalized patients had an olfactory or taste disorder.^4^ SARS-CoV-2 can be transmitted in the asymptomatic or paucisymptomatic stages; therefore, olfactory and taste disorders can be significant signs for its early detection to control transmission. We found that olfactory and taste disorders can be the first and only signs of COVID-19 pneumonia.

A 42-year-old man was admitted to the Soonchunhyang University Seoul Hospital in the Republic of Korea (ROK) for isolation and care for COVID-19 on March 26, 2020. He had been self-quarantined for 14 days since March 12 due to close contact with a confirmed SARS-CoV-2–positive patient, who was his cohabitant. In ROK, close contacts are tested for SARS-CoV-2 after 14 days of quarantine to exclude asymptomatic infections. Although he had no clinical symptoms or signs of COVID-19 such as fever, myalgia, cough, and sore throat, on March 26 (the final day of his quarantine) he was confirmed positive based on a polymerase chain reaction (PCR) test (Rdrp gene, cycle threshold value of 30.28 on sputum and 33.47 on nasopharyngeal and oropharyngeal swab). The governmental investigation team considered this an asymptomatic infection. However, the patient had developed problems with smell and taste simultaneously on March 14, after 2 days of quarantine. He did not have rhinorrhea or nasal obstruction but complained of a metallic taste in his mouth. The symptoms persisted for >2 weeks. We graded the olfactory and taste disorder on a visual analog scale of 0 to 10 (0 anosmia and 10 no olfactory or taste disorder). On quarantine day 3 (March 14), he had grade 5 symptoms, which improved by quarantine day 14 (March 26). On day 4 of hospitalization, we performed chest computed tomography (CT), which showed unilateral patchy or nodular ground-glass opacities and airspace consolidations in the right-middle and right-lower lobes.

This case of a SARS-CoV-2–positive patient with radiologically proven pneumonia on chest CT, who presented with only olfactory and taste disorders and no other clinical manifestations, suggests that previous cases with asymptomatic infections could have been misclassified. Considering the viral load in our patient, which was measured after 14 days of quarantine, SARS-CoV-2–positive patients, even when paucisymptomatic, could have relatively high viral titers, which could contribute to the rapid transmission of SARS-CoV-2.^5,6^ Moreover, because transmission can occur in the early course of infection, identification of such initial symptoms can help with the early detection of SARS-CoV-2. With this report, we emphasize the necessity for more intensive screening criteria for SARS-CoV-2 infections to ensure their appropriate identification and the prompt quarantine of suspected patients to help prevent the transmission of this virus.
